# Crizotinib in patients with anaplastic lymphoma kinase-positive advanced non-small cell lung cancer versus chemotherapy as a first-line treatment

**DOI:** 10.1186/s12885-017-3720-8

**Published:** 2018-01-03

**Authors:** Jianya Zhou, Jing Zheng, Xiaochen Zhang, Jing Zhao, Yanping Zhu, Qian Shen, Yuehong Wang, Ke Sun, Zeying Zhang, Zhijie Pan, Yihong Shen, Jianying Zhou

**Affiliations:** 1 Department of Respiratory Disease, Thoracic Disease Center, The First Affiliated Hospital, College of Medicine, Zhejiang University, No. 79, Qingchun Road, Xiacheng District, Hangzhou, China; 2 Department of Medical Oncology, The First Affiliated Hospital, College of Medicine, Zhejiang University, No. 79, Qingchun Road, Shangcheng District, Hangzhou, China; 3 Department of Pathology, The First Affiliated Hospital, College of Medicine, Zhejiang University, No. 79, Qingchun Road, Shangcheng District, Hangzhou, China

**Keywords:** Crizotinib, ALK, Non-small cell lung cancer, Efficacy, Pemetrexed

## Abstract

**Background:**

To compare the efficacy of crizotinib, pemetrexed and other chemotherapy regimens as a first-line treatment in patients with anaplastic lymphoma kinase (ALK)-positive non-small cell lung cancer (NSCLC) in real world clinical use and to evaluate the +86–571-87,236,876 predictive clinical factors of the efficacy of crizotinib.

**Methods:**

The 73 patients with ALK-positive advanced NSCLC were divided into three groups based on the first-line treatment: first-line crizotinib group (1-CRZ group, *n* = 32); first-line platinum-based pemetrexed treatment group (1-PP group, *n* = 28), and first-line chemotherapy platinum-based non-pemetrexed group (N1-PP, *n* = 12). Sixty eight of the 73 patients received crizotinib treatment and followed up in our hospital. Differences in the objective response rate (ORR), disease control rate (DCR) and progression-free survival (PFS) were compared in the different groups. The clinical factors were evaluated to predict the efficacy of crizotinib by the *Kaplan–Meier* survival analysis and Cox proportional hazards model.

**Results:**

The PFS, ORR, DCR were 16.1 months, 78.1% (25/32) and 100% (32/32) in the 1-CRZ group; were 6.0 months, 17.9% (5/28) and 57.2% (16/28) in the 1-PP group; and were 2.9 months, 15.4% (2/13) and 46.2% (6/13) in the N1-PP group. The PFS of the 1-CRZ group was significantly longer than that of the 1-PP group (*P* < 0.001) and the N1-PP group (P < 0.001). The ORR and DCR of the 1-CRZ group was significantly greater than that of the 1-PP group and the N1-PP group (all the P < 0.001). Higher Eastern Cooperative Oncology Group (ECOG) performance status score (> = 2) (HR 2.345, 95% CI 1.137–4.834, *P* = 0.021) and patients received crizotinib after N1-PP chemotherapy (HR 2.345, 95% CI 1.137–4.834, P = 0.021) were two factors associated with shorter PFS after crizotinib treatment.

**Conclusions:**

In patients with ALK-positive NSCLC who did not receive previous treatment, crizotinib was superior to standard chemotherapy for the longer PFS and greater ORR and DCR. Higher ECOG score (> = 2) and patients received crizotinib after N1-PP chemotherapy predict poor efficacy of crizotinib.

## Background

Lung cancer is the leading cause of cancer-related death worldwide with an estimated 1.4 million deaths per year [1]. Traditionally, lung cancer has been histologically divided into non-small cell lung cancer (NSCLC) and small cell lung cancer. Approximately 85–90% of all lung cancer cases are carcinomas of NSCLC [1, 2]. The development of epidermal growth factor receptor (EGFR)-targeted tyrosine kinase inhibitors (TKI) led to a different molecular pathology classification in terms of targeted therapies for lung cancer.

In 2007, the echinoderm microtubule-associated protein-like 4-anaplastic lymphoma kinase (EML4-ALK) rearrangement was discovered in NSCLC. Overall, *ALK* rearrangements are found in approximately 3% to 7% of patients with NSCLC but have been identified primarily in lung adenocarcinomas and are more frequently found in younger patients and in never or light smokers [3–6].

*ALK* rearrangements in NSCLC were associated with prolonged progression-free survival (PFS) of patients who received pemetrexed-based chemotherapy before the discovery of crizotinib, a targeted ALK tyrosine kinase inhibitor [7, 8]. Crizotinib is an oral multitargeted inhibitor of receptor tyrosine kinases including ALK, tyrosine-protein kinase Met (c-Met, also known as hepatocyte growth factor receptor, HGFR), and Recepteur d’Origine Nantais (RON, also known as Macrophage-stimulating protein receptor, MST1R) [9, 10]. Crizotinib has demonstrated concentration-dependent inhibition of ALK and c-Met phosphorylation in cell-based assays of tumour cell lines and has also demonstrated antitumor activity in mice with tumour xenografts that express ALK fusion proteins or c-Met [10, 11]. The antitumour efficacy of crizotinib was confirmed in several clinical trials for patients with advanced cancers with ALK rearrangements who either were or were not treated as a first-line therapy [11–13]. Not only was the tumour assessment-based outcome improved but the patient-reported outcome (PRO) was also improved in patients who were enrolled in these clinical trials. The dramatic effectiveness [11–14] and the tolerable side effects [14–16] observed in these clinical trials were the impetus for accelerated approval of crizotinib by the US Food and Drug Administration in 2011.

Approval of crizotinib by the China Food and Drug Adiministration was given in Jan 2013. Although some Chinese patients bought crizotinib from overseas before the approval, many more Chinese patients with advanced ALK-positive lung cancer received crizotinib treatment after its approval. We observed 73 patients with advanced ALK-positive lung cancer who underwent different treatment strategies, and 93.2% (68/73) of the patients received crizotinib treatment. Here, we compare the efficacy of crizotinib, pemetrexed and other chemotherapy regimens as a first line treatment of Chinese patients with ALK-positive NSCLC and evaluate the impact of first line therapy (pemetrexed or not-pemetrexed), brain metastasis (BM) before crizotinib treatment, ECOG score etc. on the PFS of crizotinib treatment by the *Kaplan–Meier* survival analysis and *Cox proportional hazards model* in a real world.

## Methods

### Patient selection and grouping

Approval for this study was obtained by Ethics Committee of the first affiliated hospital of Zhejiang University and the ethics committee waived the use of the inform consent. All 73 patients met the following conditions: 1. *ALK* gene rearrangements in tumour biopsies obtained from these patients were confirmed by fluorescence in situ hybridization (FISH) using the Vysis ALK break-apart probe set (Abbott Laboratories, Abbott Park, IL, USA). Positive cells were defined as: red and green signals that were separated by ≥ 2 signal diameters or deleted 5’ALK green signal observed in tumour cell nuclei. FISH-positive cases were classified as a more than 15% percentage of the total positive cells [17]; 2. When the patients were diagnosed, TNM staging was determined to be IIIB or IV according to the seventh edition of the Union for International Cancer Control and American Joint Committee on Lung Cancer TNM classification. 3. The time from diagnosis to the cut-off date of our study (31-OCT-2016) was at least one year. 4. All patients received chemotherapy or crizotinib treatment and were followed-up in our hospital.

### Patient grouping

According to their treatment history retrospectively obtained from the medical record, all patients were divided into three groups according to first-line treatment: first-line crizotinib group (1-CRZ group, *n* = 32); first-line platinum-based pemetrexed treatment group (1-PP group, *n* = 28); first-line chemotherapy platinum-based non-pemetrexed group (N1-PP, *n* = 13).

### Data collection

All patients were followed-up from the day when ALK rearrangement was confirmed until 31-OCT-2016. The interval systemic imaging (computed tomography [CT] or positron emission tomography/CT [PET/CT]) were obtained at the physician’s discretion for tumour assessment. Tumour assessment was performed according to the Response Evaluation Criteria in Solid Tumors 1.1. The baseline epidemiological data, gender, age, smoking history, Eastern Cooperative Oncology Group (ECOG) performance status score, extent of disease, treatment history, and response to therapy were retrospectively extracted from the medical record of each patient retrospectively.

The objective response rate (ORR) was defined as the sum of the rates of disease partial responses (PR) plus complete responses (CR). The disease control rate (DCR) was defined as the sum of the rates of PR, CR and stable disease (SD). Both the ORR and the DCR were calculated in our study. Progression-free survival (PFS) was defined as the time from the treatment with crizotinib or chemotherapy to progressive disease (PD) or death.

### Statistical analysis

Differences in the categorical data, such as smoking history, ECOG score, ORR and DCR among the different groups were compared using the *Chi-Square* test. The differences in age were compared by the *Kruskal-Wallis* test. The *Kaplan–Meier* method was used to estimate PFS and the difference of different groups were compared by using *log-rank* test. Covariates with a *P* ≤ 0.10 in univariate analysis were included in the multivariate model. Multivariate analysis was performed by using the *Cox proportional hazards model*.

## Results

### Demographics

All 73 patients were diagnosed with unresectable advanced ALK-positive lung cancer and were treated in our hospital. The mean follow-up was 30.2 months (range: 12–57 months). Among all the 73 patients, 68 patients received crizotinib therapy as first-line or not first line treatment. Three patients were excluded for the PFS data was not evaluable (NE) and the other 65 patients included 31 patients from the 1-CRZ group, 22 patients from 1-PP group and 12 patients from N1-PP group.

In all, 50.7% (37/73) of the patients were female, and only 32.9% (24/73) of the patients were current or former smokers; the mean age of the patients was 51.4 (range: 23–73) years old. The baseline epidemiological characteristics at the time of diagnosis including gender, smoking history, ECOG score, and extent of disease were similar among patients in the 1-PP, N1-PP and 1-CRZ groups (Table 1). Patients in the N1-PP group were younger than the other two groups (*P* = 0.045). In addition, the difference of the epidemiological characteristics except for ECOG score at the time of crizotinib treatment among patients from different groups was also not significant (Table 2). 33.3% (4/12) patients from N1-PP group had ≥ 2 ECOG score, which was more than patients from 1-CRZ group (6.5%, *P* = 0.042) and similar with patients from 1-PP group (18.2%, *P* = 0.410).

**Table 1:** Comparison of the baseline epidemiological characteristics of the different groups

Characteristics	Group				*P* value
	Total	1-CRZ	1-PP	N1-PP	
Total, n	73	32	28	13	
Gender, n (%)					
Male	36 (49.3)	19 (59.4)	11 (39.3)	6 (46.2)	0.290
Female	37 (50.7)	13 (40.6)	17 (60.7)	7 (53.8)	
Median age, year (range)	51.4 (23–73)	53.3 (30–73)	49.6 (23–71)	43.2 (23–61)	0.045
Smoking, n (%)					
Never	49 (67.1)	19(59.4)	21 (75.0)	9 (69.2)	0.431
Current/Former	24 (32.9)	13 (40.6)	7 (25.0)	4 (30.8)	
ECOG score, n (%)					
0 or 1	69 (94.5)	30 (93.8)	26 (92.9)	13 (100.0)	0.625
> = 2	4 (5.5)	2 (6.2)	2 (7.1)	0 (0)	
Extent of disease, n (%)					
Locally advanced	14 (19.2)	6 (18.8)	5 (17.9)	3 (23.1)	0.922
Metastatic	59 (80.8)	26 (81.2)	23 (82.1)	10 (76.9)	

Note. — Unless otherwise indicated, the data are shown as numbers with percentages in parentheses. The statistical analysis for age was performed using the Mann–Whitney test. The statistical analyses for other clinical features were performed using the *Chi-Square* test

**Table 2:** Comparison of the epidemiological characteristics for patients received crizotinib treatment

Characteristics	Group				P value
	Total	1-CRZ	1-PP	N1-PP	
Total, n	65	31	22	12	
Gender, n (%)					
Male	31 (47.7)	18 (58.1)	8 (36.4)	5 (41.7)	0.267
Female	34 (52.3)	13 (41.9)	14 (63.6)	7 (58.3)	
Median age, year (range)	50.2 (23–73)	50.9 (30–73)	51.9 (23–71)	42.5 (23–61)	0.640
Smoking, n (%)					
Never	44 (67.7)	19 (61.3)	17 (77.3)	8 (66.7)	0.470
Current/Former	21 (32.3)	12 (38.7)	5 (22.7)	4 (33.3)	
ECOG score, n (%)					
0 or 1	55 (84.6)	29 (93.5)	18 (81.8)	8 (66.7)	0.035
> = 2	10 (15.4)	2 (6.5)	4 (18.2)	4 (33.3)	
Extent of disease, n (%)					
Locally advanced	10 (15.4)	5 (16.1)	3 (13.6)	2 (16.7)	0.961
Metastatic	55 (84.6)	26 (83.9)	19 (86.4)	10 (83.3)	

Note. — Unless otherwise indicated, the data are shown as numbers with percentages in parentheses. The statistical analysis for age was performed using the Mann–Whitney test. The statistical analyses for other clinical features were performed using the *Chi-Square* test

### Comparison of the efficacy of different first-line treatments based on a tumour assessment

Until the cut-off date, one patient lost contact after PR in 1-CRZ group and the PFS data was not evaluable (NE); in the 1-PP group, 3 patients withdrawal chemotherapy for the side-effects and the response and PFS was NE, 2 patients asked for crizotinib treatment despite SD after chemotherapy and the PFS data was NE; one patients withdrawal chemotherapy for the side-effects and the response and PFS was NE in the N1-PP group.

The response to first line crizotinib/chemotherapy is shown in Table 3. In the 1-CRZ group, the ORR and DCR were 78.1% (25/32) and 100% (32/32); in the 1-PP group, the ORR and DCR were 17.9% (5/28) and 57.2% (16/28); the ORR and DCR were 15.4% (2/13) and 46.2% (6/13) in the N1-PP group. The ORR of patients in the 1-CRZ group was significantly greater than that of patients in the 1-PP group (*P* < 0.001) and the N1-PP group (*P* < 0.001). The DCR of patients in the 1-CRZ group was significantly greater than that of patients in the 1-PP group (*P* < 0.001) and N1-PP group (P < 0.001). Both the ORR and DCR were similar between the 1-PP group and the N1-PP group (*P* = 1.000, 0.737, respectively).

**Table 3:** Efficacy comparison of different first-line treatments based on a tumour assessment

Tumour assessment	First-line treatment			P value
	1-CRZ(n = 32)	1-PP(n = 28)	N1-PP(n = 13)	
Type of response, n %				
Complete response	1 (3.1)	0 (0)	0 (0)	
Partial response	24 (75.0)	5 (17.9)	2 (15.4)	
Stable disease	7 (21.9)	11 (39.3)	4 (30.8)	
Progressive disease	0 (0)	9 (32.1)	6 (46.1)	
Not evaluable	0 (0)	3 (10.7)	1 (7.7)	
ORR(%)	78.1	17.9	15.4	<0.001
DCR(%)	100.0	57.2	46.2	<0.001

Note. — Unless otherwise indicated, the data are shown as numbers with percentages in parentheses. The statistical analysis for DCR and ORR were performed using the *Chi-Square* test

**Fig. 1:**
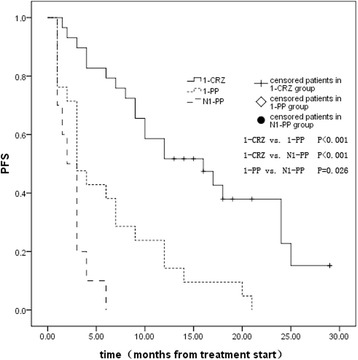
First-line treatment PFS of patients in the 1-CRZ group, the 1-PP group

The PFS of the patients in the 1-CRZ group (*n* = 31), 1-PP group (*n* = 23), and N1-PP group (*n* = 12) is shown in Fig. 1. The median PFS of the patients in the three groups was 16.1 months (95% confidence interval [CI], 12.7 to 19.4), 6.0 months(95% CI, 3.6 to 8.4), and 2.9 months (95% CI, 1.6 to 4.1), respectively. The PFS of patients in the 1-CRZ group was significantly longer than that of patients in the 1-PP group (P < 0.001) and the N1-PP group (P < 0.001). The PFS of patients in the 1-PP group was not significantly longer than that of the patients in the N1-PP group (*P* = 0.056).

Overall survival (OS) calculations were incomplete for only 28.8% (21/73) patients in our study had died by the cut-off date.

### Impact of first line therapy on the PFS of crizotinib treatment

**Fig. 2:**
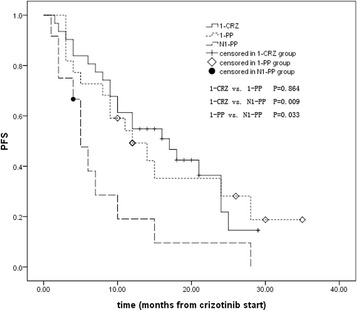
Crizotinib PFS of patients from the 1-CRZ group, the 1-PP group and the N1-PP group

The PFS after crizotinib treatment among patients from different groups were shown in Fig. 2. The PFS of patients from the 1-CRZ group (16.1 months, 95%CI, 12.7 to 19.4) and from the 1-PP group (16.2 months, 95%CI 10.9 to 21.5) were both significantly longer than PFS of patients from N1-PP group (8.0 months, 95%CI, 3.3 to 12.7, *P* = 0.009, 0.033,respectively).

### Brain metastasis (BM) in patients received crizotinib treatment

**Fig. 3:**
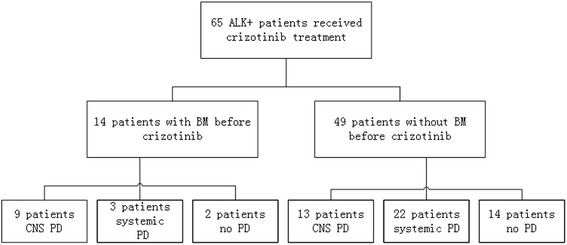
The disease progression pattern for all patients received crizotinib treatment

Among all the 65 crizotinib treated patients, 14 (21.5%) patients had BM before crizotinib treatment and all received intracranial treatment such as stereotactic radiotherapy (SRT), whole brain radiotherapy (WBRT) or surgeon except one patient. The disease progression pattern for all patients was shown in Fig. 3. The central nervous system (CNS) was the initial progression site in 22 (46.8%) patients among the 47 patients evaluated PD at the cut-off date. Patients with BM before crizotinib therapy were more easily present CNS progression (9/14, 64.3%) than patients without BM before crizotinib intake (13/51, 25.5%, *P* = 0.006). The PFS of crizotinib in patients without BM (16.3 months) was longer than in patients with BM before crizotinib treatment (11.6 months), however the difference was not significant (*P* = 0.123).

### Multivariate analysis of predictive clinical factors of PFS after crizotinib treatment

The univariate analysis demonstrated that higher ECOG score (> = 2) and patients from N1-PP group had shorter PFS and the result was confirmed by multivariate analysis (Table 4). Higher ECOG score (> = 2) was associated with shorter PFS after crizotinib treatment (HR 2.345, 95% CI 1.137–4.834, *P* = 0.021). The result was also accordant with PFS analysis, patients with higher ECOG score (> = 2) showed shorter PFS (8.5 months) than that of patients with 0 or 1 ECOG score (16.7 months, *P* = 0.007)**.** Patients received crizotinib after N1-PP chemotherapy were another factors predict shorter PFS than patients received crizotinib after 1-PP chemotherapy or as first-line treatment (HR 2.335, 95% CI 1.162–4.691, *P* = 0.017).

**Table 4:** Predictive clinical factors of PFS after crizotinib treatment

Clinical factors	Univariate analysis P value	Multivariate analysis		
		HR	95%CI	P value
Gender (male versus female)	0.318	–	–	–
Age	0.175	–	–	–
Smoking history (smoker versus never smoker)	0.255	–	–	–
ECOG score (> = 2 versus 0 or 1)	0.011	2.345	1.137–4.834	0.021
TNM stage (IV versus IIIB)	0.600	–	–	–
BM status (with versus without BM before crzotinib)	0.137	–	–	–
First line treatment (N1-PP versus 1-PP or crizotinib)	0.009	2.335	1.162–4.691	0.017

Note. —Covariates with a P ≤ 0.10 in univariate analysis were included in the multivariate model. Multivariate analysis was performed by using the *Cox proportional hazards model*

Other variables, such as age, smoking history, BM existence before crizotinib, were all not predictive factors of PFS after crizotinib treatment.

## Discussion

Our study compared the efficacy of crizotinib, pemetrexed and other chemotherapy regimens as first line treatments in patients with ALK-positive NSCLC and estimated the efficacy and predictive clinical factors of crizotinib in real world clinical use. Our study indicated the superiority of first line crizotinib treatment over standard chemotherapy in patients with advanced ALK-positive NSCLC as they had a significantly longer PFS and greater DCR and ORR. We also demonstrated that higher ECOG score and received N1-PP chemotherapy before crizotinib were the independent risk factors of short PFS of crizotinib. Patients with BM before crizotinib seemed more easily to occur CNS progression than patients without BM, however, the existence of BM was not related to the PFS of crizotinib in our study.

The efficacy of pemetrexed-based chemotherapy in NSCLC patients with ALK rearrangement was confirmed in several studies, as both a higher response rate and a prolonged PFS were observed [7, 8, 14]. However, the benefit of pemetrexed is less than that of crizotinib, as shown in clinical trials and in our study. The priority of crizotinib as a first-line treatment in our study was consistent with the results of a phase 3 clinical trial [18]. Benjamin et al. [18] reported better efficacy of first-line crizotinib treatment compared with pemetrexed-based chemotherapy (median PFS, 10.9 months vs. 7.0 months; ORR, 74.0% vs. 45.0%) in patients with advanced ALK-positive non-squamous NSCLC who had received no previous systemic treatment.

The efficacy of crizotinib as a first-line setting or after chemotherapy was quite different and the phenomenon was observed in several studies. Camidge et al. [11] revealed that in a phase 1 study, the PFS was 18.3 months in patients (*n* = 24) who received first-line crizotinib treatment and was 9.2 months in patients (*n* = 125) who received crizotinib as a second-line or later treatment. As the results of two phase 3 trials, Benjamin et al. [17] reported that the median PFS of patients who were treated with crizotinib as a first-line treatment was 10.9 months; however, Shaw et al. [13] reported that the PFS was 7.7 months in ALK-positive patients who had received one prior platinum-based regimen. Our study demonstrated the similar result and provided more information about the phenomenon. Patients received crizotinib as a first line treatment or after pemetrexed chemotherapy had a significantly longer crizotinib PFS than patients received crizotinib after non-pemetrexed chemotherapy in our study.

On the whole, 1-PP as first line treatment in ALK-positive patients had a shorter PFS than crizotinib, however, the use of 1-PP did not affect subsequent crizotinib efficacy. N1-PP therapy not only had worse efficacy of the first-line treatment but also affect the subsequent efficacy of crizotinib. Patients after N1-PP usually with worse performance score (ECOG score) might be one reason of the worse efficacy of crizotinib. Our results indicated that crizotinib as a first-line therapy or after pemetrexed chemotherapy in patients who were positive for ALK-rearrangement might have maximized the probability that these patients would benefit from ALK-directed therapy. N1-PP chemotherapy was not recommended for ALK+ NSCLC patients.

CNS progression in ALK rearranged NSCLC patients treated with crizotinib appeared to have a great incidence in patients with or without BM before crizotinib treatment. CNS progression accounted for 46.8% (22/47) all the PD patients in our study. The incidence was similar with Yoshida et al.’s study (50%, 24/48) [19]. Poor penetration rate of crizotinib to the cerebrospinal fluid (CSF) may be the the main reason of the great CNS progression incidence. Several cases were reported that the crizotinib CSF concentration was very low with a CSF-to-plasma ration of 0.006–0.0026 [20, 21]. Yoshida et al. also reported that there was a significantly shorter median PFS in the BM versus the non-BM patients before crizotinib treatment (median PFS: 6.7 months vs. 10.2 months, *P* = 0.0347) [19]. In their study, multivariate analysis revealed untreated BM were associated with the PFS duration (HR 2.314, 95% CI 1.153–4.400, *P* = 0.0196). Our study also revealed that BM status was significantly associated the occurrence of CNS progression, however, the PFS between patients with and without BM before crizotinib treatment were not significant. We deem it was because most of the patients (92.9%, 13/14) with BM in our study received intracranial treatment, such as WBRT or SRT.

## Conclusions

In conclusion, in patients with ALK-positive NSCLC who did not receive previous treatment, crizotinib was superior to standard chemotherapy and was associated with a longer PFS and a greater DCR and ORR. Multivariate analysis revealed that higher ECOG score and received N1-PP chemotherapy before crizotinib were the independent risk factors of short PFS of crizotinib. N1-PP chemotherapy was not recommended for ALK+ NSCLC patients for it not only had shorter PFS as first-line treatment but also affect the subsequent efficacy of crizotinib. Patients with BM before crizotinib were more easily to occur CNS progression than patients without BM, however, BM after appropriate intracranial therapy was not associated with the PFS of crizotinib.

## Abbreviations

1-PP: First-line platinum-based pemetrexed treatment
ALK: Anaplastic lymphoma kinase
BM: Brain metastasis
c-Met: Tyrosine-protein kinase Met
CNS: Central nervous system
CR: Complete responses
CRZ: First-line crizotinib
CT: Computed tomography
DCR: Disease control rate
ECOG: Eastern Cooperative Oncology Group
EGFR: Epidermal growth factor receptor
EML4-ALK: Echinoderm microtubule-associated protein-like 4-anaplastic lymphoma kinase
HGFR: Hepatocyte growth factor receptor
MST1R: Macrophage-stimulating protein receptor
N1-PP: First-line chemotherapy platinum-based non-pemetrexed
NSCLC: Non-small cell lung cancer
ORR: Objective response rate
OS: Overall survival
PD: Progressive disease
PET: Positron emission tomography
PFS: Progression-free survival
PR: Partial responses
PRO: Patient-reported outcome
RON: Recepteur d’Origine Nantais
SD: Stable disease
SRT: Stereotactic radiotherapy
TKI: Tyrosine kinase inhibitors
WBRT: Whole brain radiotherapy
